# Community oriented primary care in Tshwane District, South Africa: Assessing the first phase of implementation

**DOI:** 10.4102/phcfm.v5i1.477

**Published:** 2013-04-02

**Authors:** Hans-Friedemann Kinkel, Tessa Marcus, Shehla Memon, Nomonde Bam, Jannie Hugo

**Affiliations:** 1 Department of Family Medicine, University of Pretoria, South Africa; 2 Foundation for Professional Development, Pretoria, South Africa

## Abstract

**Background:**

Re-engineering primary health care is a cornerstone of the health sector reform initiated nationally in South Africa in 2009. Using the concept of ward based NGO-run health posts, Tshwane District, Gauteng, began implementing community oriented primary care (COPC) through ward based outreach teams (WBOT) in seven wards during 2011.

**Objectives:**

This study sought to gain insight into how primary health care providers understood and perceived the first phase of implementing COPC in the Tshwane district.

**Method:**

Qualitative research was performed through focus group interviews with staff of the seven health posts during September 2011 and October 2011. It explored primary health care providers’ understanding, perception and experience of COPC.

**Results:**

Participants raised organisational, workplace and community relationship issues in the discussions. Organisationally, these related to the process of initiating and setting up COPC and the relationship between governmental and nongovernmental organisations. Issues that arose around the workplace related to the job situation and employment status and remuneration of health post staff. Community related issues centred on the role and relationship between service providers and their communities.

**Conclusion:**

COPC touched a responsive nerve in the health care system, both nationally and locally. It was seen as an effective way to respond to South Africa's crisis of health care. Initiating the reform was inevitably a complex process. In this initial phase of implementing COPC the political commitment of governmental and nongovernmental organisations was evident. What still had to be worked through was how the collaboration would materialise in practice on the ground.

## Abstrait

**Communauté des soins axés primaire dans le district de Tshwane, Afrique du Sud: évaluation de la première phase de mise en œuvre**

**Présentation:**

La restructuration des soins de santé primaire a constitué une étape clé de la réforme du secteur de la santé initiée en Afrique du Sud au niveau national en 2009. Utilisant le concept de postes de santé gérés par des ONG et basés dans les quartiers, le district de Tshwane, dans la province du Gauteng, a commence à mettre en œuvre des soins de santé primaire communautaires (COPC, *Community oriented primary care*) dans sept quartiers au cours de l'année 2011.

**Objectifs:**

L'objectif de cette étude était d'obtenir des informations sur la manière dont les fournisseurs de soins de santé primaire comprenaient et percevaient la première phase de mise en œuvre des COPC dans le district de Tshwane.

**Méthode:**

Une étude qualitative a été réalisée par le biais de groupes de discussion avec le personnel de sept postes de santé au cours des mois de septembre et d'octobre 2011. Celle-ci a permis d’étudier la compréhension, la perception et l'expérience qu'avaient les fournisseurs de soins de santé des COPC.

**Résultats:**

Au cours des discussions, les participants ont soulevé des questions d'ordre organisationnel, relatives au lieu de travail et aux relations communautaires. Sur le plan organisationnel, ces questions se rapportaient au processus d'initiation et de mise en place des COPC et à la relation entre les organisations gouvernementales et non gouvernementales. Les questions associées au lieu de travail se rapportaient à la situation professionnelle et au statut professionnel et à la rémunération du personnel des postes de santé. Les questions associées à la communauté étaient centrées sur le rôle et la relation entre les prestataires de services et leurs communautés.

**Conclusion:**

Les COPC on permis de toucher un point sensible du système de santé, tant au niveau national que local. Ils étaient perçus comme un moyen efficace de répondre à la crise des services de santé à laquelle l'Afrique du Sud était confrontée. Au cours de cette première phase de mise en œuvre des COPC, l'engagement politique des organisations gouvernementales et non gouvernementales est apparu de manière évidente.

## Introduction

Municipal ward based primary health care, also referred to as community oriented primary care (COPC), is a critical component of a national effort to reform the health sector in South Africa.^1, 2, 3, 4^ Received favourably on a political level, this policy reform has been taken up in the Tshwane district of Gauteng as well as elsewhere in South Africa. In Tshwane it has been operationalised through the creation of health posts. Located in communities, health posts serve defined populations in defined geographic areas within specific municipal wards. The health post is conceptualised as a ‘nerve centre’ that drives and coordinates all community based primary health care interventions. It is the point of departure for taking health promotion, prevention and early detection of diseases, treatment support and rehabilitation out to individuals and families in defined communities and the point of entry for bringing people and their families into the health and social care systems. In Tshwane health posts have been established at and in collaboration with nongovernmental organisations (NGOs). They are funded by government and have an established local presence in communities through community health workers (CHWs) who deliver existing vertical programmes such as TB, HIV or home based care.

No matter the endeavour, the translation of an idea into practice is the acid test of making something come to life. This process is no less of a challenge in health care than in manufacturing or creativity. In policy terms, the jargon used for this part of the process is called implementation. Whilst considerable time is often devoted to policy framing in precise and accurate terms, the detailed negotiation and collaborative work that has to go into implementation is often underestimated or even neglected.^5^ In general, as the nature and impact of an initiative becomes more evident it gives rise to a multiplicity of issues. These come from a variety of sources, particularly the extent to which the ground for the reform has been prepared and practical details have been worked through, and the responses and reactions of people, both those who are expected to implement the reform as well as the intended and unintended beneficiaries.^5^ This means that responses to a reform can't be predetermined, even when considerable care is taken in policy formulation and despite its initiator's best intentions. Generally reform invokes a mixed response, creating both obstacles to and opportunities for successful implementation.

### Key focus

This paper seeks to understand how primary health care providers at municipal ward based health posts interpret the concept of COPC, the challenges they face in implementing the reform, and the nature of their concerns and worries in order to reflect on both the obstacles to and opportunities for successful implementation and to learn from practice.

### Background

The population of South Africa is in poor health. Life expectancy at birth has dropped dramatically, falling from an average of 61 years in 1990 to 51 years in 2009.^6^ This significant decline is intimately connected to the HIV and TB epidemics. Maternal and child mortality has increased and remains persistently high, at 625 deaths per 100 000 live births (maternal mortality) and 104 deaths per 1000 live births (under 5 mortality).^7^ There is extreme inequality (Gini Coefficient of income inequality = 0.70 in 2008) and widespread poverty (3 in 10 people live below $2/day 2008).^8, 9^ Inequality and poverty together lead to undernourishment and malnutrition and give rise to ill health and disability. They are also intimately linked to chronic diseases that are on the increase across all social segments of the population.^10, 11, 12^ Many of these poor health indicators are typical of low income countries, yet South Africa is a middle income country that makes significant allocations to health care (8.5% of GDP) and has good health care coverage.^6^ This situation has been described as the health paradox of South Africa.^13^

A substantial part of the answer to the cause of the health paradox lies in the structure and functioning of the existing health care system.^14, 15^ It is built on unequal, parallel streams that poorly interface with one another. It gravitates towards high cost, high tech interventions that make poor use of limited skills and seriously constrained resources at all levels of the system. It is a system that leads to growing health inequities that compound social inequality, as people are increasingly forced to incur out-of-pocket expenses in their search for adequate and quality care.^16^ Overall it is a system that fails to meet the basic health care needs of the majority of the population.^17^

Recognising the critical nature of the status quo in the health sector, the South African government is driving wide ranging health sector reforms.^1, 2, 3, 18^ The re-engineering of primary health care is central to the reforms.^2, 3^ A recent policy paper by the National Department of Health (NDOH)^3^ outlines this intent in more detail:

Primary health care services will be re-engineered to focus mainly on health promotion, [*and*] preventive care whilst also ensuring that quality curative and rehabilitative services appropriate to this level of care are rendered. […] These services will be population orientated with extensive community outreach and home based services, and in which community health workers form an essential part. (p. 23–24)

In 2010 the Gauteng Department of Health (GPDOH), Tshwane district initiated a collaborative process with the Departments of Family Medicine at the University of Pretoria and University of Limpopo and Medical University of Southern Africa (Medunsa) as well as with the Foundation for Professional Development (FPD), a local nonprofit organisation and institute of higher education, to implement a municipal ward based primary health care model. The initiative drew on the assumptions and approach of COPC. COPC was developed by Sidney and Emily Kark in the 1940s, and they initially implemented it in rural Pholela in Natal (now KwaZulu-Natal).^19, 20, 21, 22, 23, 24, 25, 26^ Subsequently they and others have used COPC as an approach to primary health care in many communities around the world.^27, 28, 29, 30, 31, 32, 33, 34, 35^ Most recently the principles of COPC (local health and institutional analysis, focused service from prevention to palliation, equity, practice with science and service integration around users) have been elaborated to support the current efforts to reorient primary health care in South Africa^36^.

In the Tshwane district COPC is designed around health posts.^37^ The posts are the structures that are physically located in communities, as well as health care practitioner teams, initially comprising professional nurses and CHWs. The responsibility of a team is to interact in a proactive way with every household (both with the collective unit and with individual members) in their jurisdiction. Their role is to promote health, prevent and/or detect disease early, and support treatment, rehabilitation and palliation, and to do this in a way that develops capacity and shared responsibility for heath care between service providers and service users.

By mid 2011 health posts had been established in seven communities in the Tshwane district. This was achieved as a result of collaborative work between the Tshwane district (GPDOH), the Department of Family Medicine of the University of Pretoria and FPD. It involved active partnering with local NGOs that were already well established and supported by and working with the Tshwane district (GPDOH). It required extensive consultation and interaction with local government and municipal ward counsellors. It also required building partnerships with information technology experts and other specialist private sector partners.

In practical terms creating health posts entailed: (i) Engaging with relevant NGOs, district and ward level counsellors as well as local clinic heads regarding COPC as an approach to primary health care. This involved introducing the concept of COPC and familiarising them with the principles and practices of the approach, as well as addressing and discussing its implications for existing practices and institutional organisation; (ii) Finding and equipping buildings or structures for the health posts; (iii) Appointing health post managers and CHW teams. For existing NGOs, this meant establishing administrative and supervisory structures to handle new staff (CHWs), who were funded through an additional funding stream; (iv) Training the health post teams which entailed the NDOH's 59–69 day training programme that all CHWs had to complete and additional training on COPC for health post managers by the Department of Family Medicine at the University of Pretoria; (v) Defining and mapping the physical boundaries of each health post; (vi) Conducting initial institutional assessments of the organisations working in each site; (vii) Appointing a person to manage COPC within the Tshwane district (GPDOH); (viii) Developing a health status assessment survey for the district; (ix) Developing a mobile information and communication application, with the assistance of a medical information technology company (Mezzanine, Medical Information Technology, Cape Town, South Africa). With this system the information collected from individuals and households can be used in real time for practical day to day activities at health post level, as well as for determining priorities, shaping intervention planning and monitoring; (x) Training the health post teams on the health status assessment (survey) and its application using mobile technology.

For the purposes of this study, the first phase of implementation is understood as the period from establishing a health post, including appointing and training of staff, until the health post becomes operational and health post staff was ready to conduct health status assessments in the community. By October 2011 seven health posts in the Tshwane district had become operational (Table 1). The second phase begins when the health post teams systematically conduct community health status assessments, prioritise health issues, develop intervention strategies and initiate interventions.

**TABLE 1:** Characteristics of the seven health posts studied by 30th September 2011: Partnering non-governmental organisations, number of households mapped per health post, number of community health workers allocated to the health post and number and percentage of community health workers who had been trained on the use of mobile devices for health status assessments.

Characteristics	HP1	HP2	HP3	HP4	HP5	HP6	HP7
Partnering NGO	yes	yes	yes	yes	yes	yes	yes
Number of households mapped	3665	4590	1552	3200	3750	3080	3000
Number of CHWs allocated to the health post†	33	8	10	26	10	10	8
Number of CHWs having received training on the use of mobile devices for health status assessments	22	8	10	26	10	10	8
Percent of CHWs having received training on the use of mobile devices for health status assessments	67%	100%	100%	100%	100%	100%	100%

NGO, non-governmental organisations; CHW, community health workers; HP, health post.

† At the time of this study not all health posts had yet been allocated their full complement of community health workers.

### Objectives

The objective of this study was to gain insight into how primary health care providers (the health post team) understood community oriented primary care and perceived the first phase of implementation.

### Contribution to field

The health care provider's understanding of a reform is crucial to its successful implementation and especially its ability to achieve its intended outcomes. This is especially true when the reform requires changes in their practices and an expansion of their skills and knowledge. By providing an understanding of the challenges and possibilities for realising the reform's objectives, this study provides insights into best practice as well as possible areas of action that can better guide future implementation.

The implementation of community oriented primary care (COPC) is an important component of health sector reform in South Africa. This work provides insight into how primary health care providers understood and perceived the first phase of implementation of COPC in the Tshwane district, South Africa. The results inform government and nongovernmental organisations about the obstacles and opportunities presented by this reform process.

### Ethical considerations

This research was approved by the Research Ethics Committee of the Faculty of Health Sciences, University of Pretoria (Protocol number 102/2011) and the GPDOH (Tshwane Research Committee).

## Research methods and design

### Design

This was a cross-sectional qualitative study based on seven focus group interviews (FGIs) that were carried out during September 2011 and October 2011 at all seven health posts in the Tshwane district.

### Materials

Prior to the focus group interviews, general information about the health post was obtained from health post managers, NGO managers and the Tshwane district COPC management team, namely, the project manager and representatives from the Tshwane district (GPDOH), the University of Pretoria, Department of Family Medicine and the Foundation for Professional Development. The duration of the focus group interviews was between 60 and 90 minutes. They were conducted on site at each of the respective health posts or their associated NGO. They each involved between five and ten participants, including CHWs and service providers who work closely with the health post, such as community development workers or community nurses from the local clinic and the health post or the NGO manager.

### Procedures

By agreement with the participants, all the sessions were conducted in English. Prospective participants were given an information leaflet about the study a week prior to the interviews. On the day of the discussion written consent was obtained from each participant before the session began.

The sessions were facilitated by two researchers who were consultants of the Department of Family Medicine, University of Pretoria and the Foundation for Professional Development. Participants were invited to discuss their understanding of COPC, the extent to which they felt they had been able to take COPC into their communities, what past practices and experiences they thought might help or hinder COPC, how they used or found ways of overcoming these, and whether they thought that they could succeed with COPC.

### Analyses

All discussions were audio-recorded and transcribed literally. Transcription occurred within three days of the interviews. The interviews were then analysed in the following manner.

The first question regarding the understanding of the concept of COPC was analysed against a list of 10 key ideas that characterize COPC and that had been addressed spontaneously by the participants, as shown in Table 2. Responses were analysed by health post.

**TABLE 2:** Health care providers’ understanding of key ideas in community oriented primary care. Asked to describe their understanding of community oriented primary care, focus group participants spontaneously addressed the key ideas shown in this table.

Key ideas in COPC	HP1	HP2	HP3	HP4	HP5	HP6	HP7
… is a delivery driven health service based on door-to-door visits	✓	✓	✓	✓	✓	—	✓
… is different from home based care	—	—	—	—	!	✓	—
… has a strong focus on health promotion, disease prevention and early disease detection	✓	✓	✓	✓	✓	✓	✓
… addresses the health of the community as a whole	✓	✓	✓	✓	!	✓	✓
… requires an understanding of the health of the community (health status assessment)	—	✓	✓	—	—	—	✓
… strives to empower communities (self-care)	✓	✓	✓	—	✓	✓	✓
… addresses the health of the whole family or household as well as the individual	—	✓	✓	✓	—	✓	✓
… aims to screen, refer and follow up individuals and families	—	✓	✓	—	✓	✓	✓
… is expected to reduce pressure in the clinics and other health facilities	✓	✓	—	✓	—	—	✓
… is based on a multi-sector approach, involving social services etc.	✓	✓	✓	—	✓	✓	✓

**Total number of key ideas expressed and understood**	**6**	**9**	**8**	**5**	**5**	**7**	**9**
**Total number of key ideas misunderstood**	**0**	**0**	**0**	**0**	**2**	**0**	**0**

COPC, community oriented primary care; HP, health post.

✓, key idea correctly understood; !, key idea not correctly understood; —, key idea not addressed.

For the remaining questions responses were aggregated across all health posts and reviewed twice. The first review sought to identify overarching themes or categories. The second review sought to attribute responses to the identified themes or categories and to refine the themes or categories more specifically.

## Discussion of results

### Stage of implementation

As Table 3 shows, all health posts were approximately at the same point of implementation by 30 September 2011, although health posts (HP) 1 and 5 had not yet completed training of all their staff.

**TABLE 3:** Stage of implementation of the seven health posts studied, by 30th September 2011. All health posts were at the same stage of implementation except HP1 and HP5, which had yet to complete health post staff training in COPC.

Implementation phase	HP1	HP2	HP3	HP4	HP5	HP6	HP7
Selecting ward or target area	✓	✓	✓	✓	✓	✓	✓
Building collaboration with ward council and local NGOs	✓	✓	✓	✓	✓	✓	✓
Identifying NGO doing community work	✓	✓	✓	✓	✓	✓	✓
Appointing health post team	✓	✓	✓	✓	✓	✓	✓
Creating shared understanding within HP team	o	✓	✓	✓	o	✓	✓
Finding health post building	✓	✓	✓	✓	✓	✓	✓
Equipping health post	✓	✓	✓	✓	✓	✓	✓
Mapping target area	✓	✓	✓	✓	✓	✓	✓
Conducting institutional assessment	✓	✓	✓	✓	✓	✓	✓
Conducting household and health assessments	—	—	—	—	—	—	—
Establishing community diagnosis	—	—	—	—	—	—	—
Prioritising problems and interventions	—	—	—	—	—	—	—
Running interventions	—	—	—	—	—	—	—
Monitoring and evaluating the interventions	—	—	—	—	—	—	—

COPC, community oriented primary care; NGO, non-governmental organisations; HP, health post.

✓, Phase completed; o, Phase not yet completed; —, Phase not yet started.

### Understanding of community oriented primary care

In all interviews the participants were able to describe spontaneously at least five of ten key ideas of COPC, although the interview at HP5 revealed some misconceptions of COPC (Table 2).

### Themes emerging in the interviews

There were three overarching themes that emerged from the review of the data – organisation, workplace and community. Issues about organisation that arose in the interviews related to the process of initiating and setting up community oriented primary care and the relationship between governmental and non-governmental organisations. Issues about the workplace that arose relate to the job situation and employment status of CHWs and the remuneration of health post staff. Issues relating to the theme of community that arose in the interviews concerned the role and relationship of the service providers to the community they serve in COPC.

### Organisation

Where the reform initiative originates often carries implications for how people feel about and respond to it. Whether the process had been devised at the top and sent down into the system or whether it had been developed in response to bottomup initiatives, in particular, carries important implications for acceptance, understanding and practice.

In 2009 the decision to re-engineer primary health care was taken at a national government level.^18^ This sparked a policy process in the Tshwane district (GPDOH) that preceded the first phase of implementation at six of the seven sites.^38^ At the seventh site, however, the 2009 national decision sparked a self initiated, NGO supported attempt to do COPC on the ground even before policy had been developed.

In Tshwane the approach to the initiative was to base COPC on a relationship with existing healthcare related NGOs located and active in the respective sites. This approach was adopted in order to maximise available infrastructure and capacity and to take advantage of existing home based care and health outreach experience as well as established links with communities where the NGOs worked.

It was clear to the Tshwane district COPC management team that the introduction of COPC would be disruptive to collaborating NGOs. Firstly, they would need to review existing approaches, practices and priorities. Whilst many were able to form the foundation of COPC, others did not fit in with the approach. Secondly, organisations would have to re-orientate and expand the issues they focus on. Thirdly they would be expected to reconsider how they work and who they serve in the community. Lastly, they would have to review their organisational structures, including reporting lines.

Although the Tshwane district COPC management team had consulted with NGOs and invited them to the process, some respondents in the focus groups said they had found this disruption disconcerting (FGI HP4: 32, 33). This was especially the case at one site where they had just completed a months-long planning process that they now felt they would have to discard in order to accommodate COPC:

‘What personally frustrated me is that we had to jump […] we were planning months in advance to get things performed and then here comes this instruction […] and we had to literally cancel whatever we were busy with, and we would [*need to*] rescheduling in order to accommodate, we did not mind, but for a future roll out I think […] there should be more flexibility’. (FGI HP4: 32)

Not surprisingly, the direction that came from the Tshwane district COPC management team at the site where COPC was selfinitiated by the local clinic and an NGO, was not seen as disruptive. Rather, they felt supported and confirmed. They also felt that with this support they were able to expand and take the initiative forward in a more effective and purposeful way (S.L., personnel communication).

In terms of the relationship between government and NGOs COPC triggered self-reflection amongst NGOs. Historically, government had encouraged NGOs to engage in community work employing CHWs.^39^ How such community activities should be carried out has been outlined in detail in a recently updated policy framework.^40^ However, NGOs that are active in community work have largely been accountable to their funders or donors rather than to the government. With COPC, the parameters of accountability shift more towards health outcomes in the communities. As a consequence there is a need for NGOs engaging in COPC to work more closely with the primary health care system. This, in turn, has implications for organisational autonomy as well as power relations as the NGOs now have to follow governmental directives. Some respondents recognised this and articulated it as a challenge that they would have to (re)negotiate with government:

There are some organizations who have been working for years […] and their systems are well in place. You know, to change things, I think it's very difficult. As a lot of people like to have the ownership and to change things is a big challenge. (FGI HP4: 41)

The relationship between government and NGOs about the conditions of employment of CHWs is an ongoing issue in the whole system.39

This notwithstanding, a collaborative partnership with the government regarding COPC remains attractive to NGOs as it fits in with their principles and promises them greater financial stability. In the focus groups respondents expressed their commitment to COPC, both as an idea and a set of practices (FGI HP1:85; HP2:78; HP3:64, 65; HP4:58; HP5:58; HP6:140; HP7:132). However, an underlying worry that they all expressed related to sustainability. On the one hand, they were concerned that the initiative may be driven by short-term political expediencies (FGI HP1:81; HP7:135):

And again, whether the government is going to sustain the program […] is a political thing. When I am a minister, I come with this suggestion and then people implement it, when she comes in, she comes with her other ideas, so some things are not sustained. So we pray that they sustain it. (FGI HP1:81)

On the other hand, they were worried about a political initiative that is not backed up financially (FGI HP1:41; HP4:63): ‘I think a lot depends on the political will, because that is where your money is coming from, to sustain it financially […]’ (FGI HP4:63).

The first phase of implementation was almost entirely supported by funding from nongovernmental sources. Whilst efforts had been made within government to secure funding that yielded formal commitment (L.M., personnel communication) actual allocations through district level budgetary processes had not yet been made at the time of writing.

### Workplace

A fundamental issue in COPC is the regularisation of employment for CHWs.39 Until now, the underlying assumption has been that home based and other community care workers work voluntarily, supported by ‘stipends’. They have therefore fallen outside the formal employment sector, a fact acknowledged by the Departments of Health and Social Development as something that has to be changed.^40^

Not surprisingly, remuneration of the CHWs was raised as a major issue of concern during the interviews. Respondents pointed to inequality in stipends between CHWs that they regarded as unfair (FGI HP2:72; HP6:59, 60; HP7:78, 117). These had come about because of different funding sources and standards of payment between and within NGOs. Respondents also pointed to late payment, which particularly affected CHWs supported by the government. Failure to transfer funding to NGOs meant that some CHWs had not been paid for up to eight months for their work (FGI HP3:47; HP7:43). Respondent worries about remuneration also centred on the absolute levels of pay and the absence of basic conditions of employment (FGI HP3:50, 51; HP4:64; HP5: 75-77). They felt that a stipend of about 1500 South African Rand is too low for them to live on. Respondents also felt that their levels of pay undermined the value of the work that CHWs do. They are expected to have completed basic training (59–69 day training paid for by the NGOs or government), to be competent in predetermined performance areas and to work a 40 hour week.

Paying CHWs stipends rather than remunerating them undermines the value of their work in their own eyes. It also undermines the value of their services to the health care system in the eyes of society. Furthermore it aggravates existing gendered discrepancies in the professions where care workers are systematically penalised because they are predominantly female, a penalty that extends to the ‘voluntary sector’.^39, 41^ For COPC, remuneration presents a practical issue of sustainability, since CHWs will be forced to seek better livelihoods outside the sector if their work continues to be undervalued. Worse still, COPC itself is undervalued and compromised if their work is undervalued, as the system is bottom-up from people through CHWs to professional nurses to clinicians and then, through them, to clinics, medical practices and hospitals.

### Community

Community, as it is understood in this study, means (i) the people who live in the community (individuals, families and groups) and (ii) the religious, cultural, political, economic and social organisations and institutions that exist or work in a particular site (schools, churches, theatres, businesses, local government etc.). Both these understandings of community are relevant to COPC.

In terms of community as people, the relationship between service providers and the people they serve is a critical component of community based care. The predominant practice of health care service has traditionally been unidirectional, i.e. from service providers to people, variously called clients or patients, and focused on cure. Conceptually, COPC challenges both these dimensions of the existing model. It shifts the attention of primary health care in the community towards health promotion, disease prevention and the early detection of illness. At the same time it changes the locus of authority for health care to a partnership built on mutual empowerment, where authority, responsibility and capacity are shared between local and external service providers and local users. Furthermore, it is about catalysing growing and self-sustaining relationships around health and care.^21, 22, 42, 43^

Basing their opinions on their experience, some respondents in the focus groups expressed doubts about the capacity of people to take responsibility for and be active partners in their own health. They described the people they serve as passive and dependent, ‘expecting handouts’ (FGI HP1:38; HP2:35). This they said had happened even when they had consciously made an effort ‘to give them health education’ (FGI HP2:35). In some cases, however, they observed that people were made passive and dependent by their extreme poverty. In these cases, it was not about expectations but that people just did not have food or the means to care for themselves (FGI HP3:33; HP5:71).

In terms of their understanding of people's willingness to accept COPC, respondents raised issues of confidentiality, privacy and stigma. Some felt that these would be an impediment to developing strong relationships, especially where CHWs and the people they served were neighbours (FGI HP6:41–44), or where CHWs were associated with HIV related organisations (FGI HP6:49; HP7:44).

Respondents also raised the issue of security. It was a worry for CHWs, who thought that they might be more vulnerable when they had to enter people's homes. Respondents also thought that because members of the communities were worried about their own security, they might be reluctant to allow CHWs into their homes.

These concerns present real challenges for primary health care as well as COPC. Trust in COPC workers and the willingness to engage in individual and collective learning, development and change in order to achieve better health for all is earned and requires both professionalism in practice and continuity in service and care.^44, 45^ It also depends on the quality of care giving, the evidence of benefits and the relationships that are forged. All of these are built into the principles of COPC, but what really matters is whether they are applied in practice.

Issues can partly be resolved by engaging with people as they arise, but they also require organisational and institutional responses. COPC confronts organisational and institutional practices within and beyond health. In the existing system, the predominant institutional model is organised around line function and a hierarchical, top-down system of accountability. Whilst collaboration is formally encouraged, systems often work against integration, shared responsibility, cooperation and responsiveness to conditions on the ground in practice.^16^

In the focus groups, respondents described various kinds of organisational and institutional blockages that they had encountered during the first phase of implementation. Some related to the origin of the authority to do COPC:

‘The people at the moment don't want to catch [*sic*] the COPC […] before they want to run it they have questions […] they want to know, “Who did this come from? The Department of Health? Or are you bringing it? Why did it not come with the city council?”’. (FGI HP7:75)

Others related to issues of hierarchy, where breach of protocol was invoked by officials because they perceived that the person introducing them to COPC was at a ‘low level’ (FGI HP6:53–55; HP7:76–77):

Marketing the programme is a challenge from our side, because we are at a certain level and you must reach other people higher than your level […]. They will tell you of the protocol and say, “Go back to your senior, your senior needs to talk to me before you.” Like for example, at the clinic here myself I couldn't go there because my seniors must meet their seniors to discuss this COPC programme before me going to them and making a partnership with them […]. (FGI HP6:53–54)

In general, respondents emphasised the importance of politicians. Above all they felt that politicians needed to be actively involved in introducing and promoting COPC for their activities to be seen as legitimate and mandated and for the reform to succeed (FGI HP2:48; HP6:92–94; HP7:104–105):

‘I think it is also important to interact with the political people. We have not met our [*ward*] councillor because he is such a busy person and we are looking very much forward to meeting him because there is nothing we can roll out without the politicians’. (FGI HP2:48)

In this they have understood the ramifications of COPC, namely that it goes beyond the health care system to the broader political, social and economic system.^46^

## Limitations of the study

The study is limited by methodology. Focus group discussions are an accepted qualitative research method. In this instance they were used to indentify emerging issues that primary care providers believe will influence the implementation of ward-based primary care in the Tshwane district. However, they are characterised by several constraints. One constraint is the potential effect they have on the ability of the participants to express themselves freely. Whilst every endeavour was made to create an atmosphere of trust in order to allow the most relevant issues to surface, there is no certainty that this was indeed the case. Another constraint is the fact that focus group discussions are context specific. The study was influenced by the time the interviews took place and the time of writing. Evolving reform is likely to raise new issues or influence the perception and relevance of the issues identified in this study. The third constraint is that the method is sensitive to interpretation bias by the researchers. More generally, as qualitative research, the findings of the study are indicative of issues rather than being generalisable.

## Recommendations

1. Primary care providers (CHWs, health post managers and NGO management) should be mobilised in the implementation of ward-based primary care. They are an important asset in ward-based primary care as they support the concept and are highly committed to establishing it in their communities.
2. There is a need to recognise that the reform carries profound implications for NGOs participating in ward-based primary care, both in terms of their organisational structures and operational practices. NGOs are willing to engage in organisational evolution if they trust the process and can be sure that it is worthwhile. Their trust will, however, only be gained if government demonstrates both political and financial commitment to ward-based primary care, including visible leadership and management, effective administration, the allocation of human resources and securing and committing sustained funding, by developing specific guidelines for these processes.
3. There is a need for all partners in primary care to be open and responsive to the uncertainty that comes with reform. As the response to the reform is often hard to predict, it is important to give it time to work itself through and make sure that unfavourable outcomes trigger a (re-) negotiation process rather rejection of the reform process.
4. A dominant theme in the study centred on the role of the CHWs. On the one hand there has been a constant problem of late or sporadic payments that undermines commitment and trust in the reform. On the other hand, the evolving role and mandate of CHWs in ward-based primary care has already led to a discussion about their professional status. Both these issues point to the urgent need to review the value of CHWs in primary care, to develop their capacity in a sustained and ongoing way and to ensure that the work they do is appropriately rewarded in accordance with labour best practices and policies and the health needs of the country that the reform seeks to address.
5. It is important that political leaders at all levels are engaged in a process of preparing communities for primary care reengineering, as their involvement directly influences the pace and effect of the reform.

## Conclusion

The idea of COPC has touched a responsive nerve in the health care system both nationally and locally. It is seen as an effective way to respond to South Africa's crisis of health. Initiating a reform is inevitably a complex process. It is rarely orderly and without complications. In this initial phase of the implementation of re-engineered primary health care the political commitment from the government and NGOs is evident. What still has to be worked through is how the collaboration will materialise in practice in the daily interfaces, both between government and NGOs at all levels and between the health posts and the communities they work in. It is expected that these issues will become clearer in the second phase of implementation, when the health post teams begin the process of assessing the health status of the communities they serve and initiate interventions.
